# Experimental data suggesting that inflammation mediated rat liver mitochondrial dysfunction results from secondary hypoxia rather than from direct effects of inflammatory mediators

**DOI:** 10.3389/fphys.2013.00138

**Published:** 2013-06-07

**Authors:** Adelheid Weidinger, Peter Dungel, Martin Perlinger, Katharina Singer, Corina Ghebes, J. Catharina Duvigneau, Andrea Müllebner, Ute Schäfer, Heinz Redl, Andrey V. Kozlov

**Affiliations:** ^1^Ludwig Boltzmann Institute for Experimental and Clinical TraumatologyVienna, Austria; ^2^Institute for Medical Chemistry, University of Veterinary Medicine ViennaVienna, Austria; ^3^Department of Neurosurgery, Medical University GrazGraz, Austria

**Keywords:** liver, reoxygenation, lipopolysaccharide, cytokine, iNOS, IL-6, HO-1, free iron

## Abstract

Systemic inflammatory response (SIR) comprises both direct effects of inflammatory mediators (IM) and indirect effects, such as secondary circulatory failure which results in tissue hypoxia (HOX). These two key components, SIR and HOX, cause multiple organ failure (MOF). Since HOX and IM occur and interact simultaneously *in vivo*, it is difficult to clarify their individual pathological impact. To eliminate this interaction, precision cut liver slices (PCLS) were used in this study aiming to dissect the effects of HOX and IM on mitochondrial function, integrity of cellular membrane, and the expression of genes associated with inflammation. HOX was induced by incubating PCLS or rat liver mitochondria at pO_2_ < 1% followed by reoxygenation (HOX/ROX model). Inflammatory injury was stimulated by incubating PCLS with IM (IM model). We found upregulation of inducible nitric oxide synthase (iNOS) expression only in the IM model, while heme oxygenase 1 (HO-1) expression was upregulated only in the HOX/ROX model. Elevated expression of interleukin 6 (IL-6) was found in both models reflecting converging pathways regulating the expression of this gene. Both models caused damage to hepatocytes resulting in the release of alanine aminotransferase (ALT). The leakage of aspartate aminotransferase (AST) was observed only during the hypoxic phase in the HOX/ROX model. The ROX phase of HOX, but not IM, drastically impaired mitochondrial electron supply via complex I and II. Additional experiments performed with isolated mitochondria showed that free iron, released during HOX, is likely a key prerequisite of mitochondrial dysfunction induced during the ROX phase. Our data suggests that mitochondrial dysfunction, previously observed in *in vivo* SIR-models, is the result of secondary circulatory failure inducing HOX rather than the result of a direct interaction of IM with liver cells.

## Introduction

Trauma, sepsis, and several types of shock are accompanied by two key pathological events. These are drastic elevation of levels of inflammatory mediators (IM), predominantly cytokines (Clarkson et al., 2005), and secondary circulatory failure causing tissue hypoxia (HOX) (Legrand et al., 2010). Systemic inflammatory response syndrome (SIRS) can be induced by either damage-associated molecular pattern molecules (DAMPs) or pathogen-associated molecular pattern molecules (PAMPs) causing non-infectious and infectious inflammatory responses that are associated with elevated IM levels. In ischemia, immune activation is mainly mediated by DAMPs, whereas in infectious diseases it is triggered by PAMPs. Cytokines, such as tumor necrosis factor alpha (TNF-α), interleukin 6 (IL-6), and interferon gamma (IFN-γ), contribute to the inflammatory response by activating pathological intracellular signaling cascades which result in cellular dysfunction and death. While TNF-α is the most potent activator of death pathways, IL-6 is one of the key regulators of the inflammatory response in the liver. It activates acute phase response, increasing the synthesis of C-reactive protein, fibrinogen, and serum amyloid A, among others. Additionally, IL-6 production has also been suggested as a common feature of ischemic injury (Kielar et al., 2005) and particularly of liver ischemia (Tacchini et al., 2006). The inducible nitric oxide synthase (iNOS) is typically upregulated by IM, predominantly by TNF-α and IFN-γ, and plays an important role in the onset of inflammatory response. Upregulation of iNOS has also been demonstrated in hepatic ischemia reperfusion models (Isobe et al., 1999). Nitric oxide (NO), a product of iNOS activity, modulates mitochondrial function and stimulates cGMP-dependent signaling. Heme oxygenase 1 (HO-1) is a member of the heat shock protein family (HSP32), catalyzing enzymatic degradation of heme, resulting in the release of equivalent amounts of biliverdin, carbon monoxide, and ferrous ion (Tenhunen et al., 1968). Upregulation of HO-1 has been shown in both conditions, inflammation (Bauer et al., 2003) and impaired circulation (Duvigneau et al., 2010). Meanwhile, it is well documented that both hypoxia/reoxygenation (HOX/ROX) and IM activate multiple transcription factors that regulate IL-6, iNOS, and HO-1 expression. However, it is less clear which condition has the major impact on the early upregulation of either gene. Furthermore, both HOX (Shiva et al., 2007) and IM (Singer et al., 2004) are often associated with impaired mitochondrial function. HOX/ischemia (Rouslin et al., 1990) as well as inflammatory response (Duvigneau et al., 2008) has been shown to decrease ATP levels. Damage to mitochondria is critical for many cellular functions, as mitochondria are involved in a variety of other processes, e.g., innate immune signaling (West et al., 2011), ROS production (Murphy, 2009) and programmed cell death (Skulachev, 1999). There is a body of literature documenting the release of alanine aminotransferase (ALT) and aspartate aminotransferase (AST) following liver ischemia (Lin et al., 2009) and SIRS (Izeboud et al., 2004). In the event of cell lysis, hepatocellular enzymes are released and can be determined in blood or in the incubation medium in *in vitro* experiments (Lerche-Langrand and Toutain, 2000). ALT is mainly present in the cytoplasm of hepatocytes whereas AST is mostly localized in mitochondria but also in the cytoplasm. Therefore, release of ALT predominantly indicates increased permeability of the cytoplasmic membrane, while the increased AST levels reflect both increased permeability of the cytoplasmic membrane and mitochondrial damage. It is difficult to differentiate *in vivo* between the pathological impact of IM and hypoxia on tissue functions because of systemic interactions. However, it is important to gain better understanding of the exact pathological mechanisms leading to organ dysfunction in order to develop efficient therapeutic strategies. Precision cut liver slices (PCLS) are a reliable *in vitro* model of liver tissue, maintaining cell–cell and cell-extracellular matrix interactions without the influence of systemic processes (Lerche-Langrand and Toutain, 2000). We applied this model to dissect the effects of IM and HOX on liver cells. We hypothesized that the above described changes in gene expression and cellular function/integrity could partially be exclusively attributed to either HOX/ROX or IM. Consequently, determination of these markers will help to understand the dynamics of disease and choose an adequate therapeutic strategy. Therefore, the main objective of this study was to investigate whether or not mitochondrial dysfunction, disintegrity of cellular membranes and the expression of genes associated with SIRS can be assigned to either HOX- or IM-dependent pathways.

## Materials and methods

### Chemicals

All reagents were obtained from Sigma-Aldrich (Vienna, Austria) unless otherwise noted.

### Animals

Adult male Sprague-Dawley rats (300–350 g) were purchased from the Animal Research Laboratories, Himberg, Austria. All animal procedures were approved by the local legislative committee and conducted according to National Institute of Health guidelines.

### White blood cells (WBC) isolation and conditioned medium generation

Eight rats were anesthetized with isoflurane (Abbott, Vienna, Austria), heparin (600 U/kg, Ebewe, Unterach, Austria) was injected and whole full blood was withdrawn from the vena cava and transferred into 50 ml flasks. Red blood cells were lysed with Schwinzer lysis buffer (0.16 M NH_4_Cl, 0.27 mM EDTA, 10 mM KHCO_3_) for 15 min at 4°C. WBC were pelleted by centrifugation (10 min, 400 g, 4°C) and washed twice in RPMI 1640 medium. Cell pellets were resuspended to 1 × 10^6^ cells/ml in RPMI 1640 medium. IM were generated by incubation of WBC with lipopolysaccharide (LPS, *E. coli* Serotype 026:B6, 6 μg/ml) for 24 h at 37°C. Finally, the cell suspension was centrifuged, the supernatant, now containing WBC-derived cytokines (conditioned medium), was pooled and stored at −80°C. The cytokine pattern of the conditioned medium determined by Myriad RBMTM (Austin, TX, USA) is shown in Table 1.

**Table 1:** **Selected cytokines present in control and conditioned medium (CM)**.

**Cytokine**	**Lower limit of detection**	**Control**	**CM**
C-reactive protein, [μg/mL]	0.017	ND^a^	0.078
Interferon γ, [pg/mL]	4.6	ND	4.6
Interleukin-1 α, [pg/mL]	36	ND	288.5
Interleukin-10, [pg/mL]	45	ND	74.7
Interleukin-11, [pg/mL	27	ND	94.5
Interleukin-2, [pg/mL]	12	ND	12
Interleukin-7, [ng/mL]	0.015	ND	0.08
Tumor necrosis factor α, [ng/mL]	0.010	ND	0.59
Monocyte chemotactic protein 1, [pg/mL]	0.996	ND	1335

a ND, not detectable.

### Preparation of PCLS

Rats were anesthetized with isoflurane and sacrificed by decapitation. The liver was extracted and placed into ice cold custodiol solution (Koehler Chemie, Bensheim, Germany) until slice preparation. Cylindrical tissue cores of 6 mm diameter were punched from the left lateral liver lobe and embedded in 3% agarose gel. Slices of 250 μm thickness were cut using a microtome (compresstome VF-300, Precisionary Instruments Inc., Greenville, NC, USA) at room temperature. Slices were incubated in ice cold Ringer solution (Fresenius Kabi, Graz, Austria) for 25 min in order to wash out intracellular compounds released due to the preparation procedure.

### Experimental models based on PCLS

In the HOX/ROX-model, HOX was induced by incubating PCLS in glass vials under nitrogen flow at pO_2_ < 1% followed by subsequent ROX with medium equilibrated with air oxygen. HOX/ROX medium was supplemented with the glycolysis inhibitor 2-deoxy-D-glucose (DOG, 10 mM). Duration of the hypoxic and ROX phases is indicated in figure legends. Normoxic control samples (NOX) were incubated in the HOX/ROX medium which was equilibrated with air during the whole incubation period. Inflammatory injury (IM-model) was simulated by incubation of PCLS in 6 well plates (Costar, Corning, USA) containing 1 mL conditioned medium. Corresponding controls were incubated in normal, not preconditioned, RPMI 1640 medium (NM) with or without LPS (6 μg/ml).

### Preparation of isolated mitochondria

Liver mitochondria were isolated by four consecutive centrifugation steps as previously described (Dungel et al., 2011). The final pellet was resuspended in buffer containing 0.25 M saccharose, 10 mM Tris, 0.5 mM ethylenediaminetetraacetic acid, and 5 mg/mL fatty acid-free bovine serum albumin (pH 7.2, 25°C). The protein concentration was analyzed using the Biuret method.

### Determination of endogenous free iron in liver sections

Liver sections were cut (2 × 2 × 10 mm) to fit to the EPR spectrometer cavity and subjected to hypoxia for 1 h under nitrogen. Sections were processed as previously described (Kozlov et al., 1992) to determine endogenous free iron following formation of nitrosyl iron complexes.

### Mitochondrial respiration

Respiratory parameters of mitochondria were monitored using a high resolution respirometer (Oxygraph-2k, Oroboros Instruments, Innsbruck, Austria). Isolated mitochondria/PCLS were incubated in buffer containing 105 mM KCl, 5 mM KH_2_PO_4_, 20 mM Tris-HCL, 0.5 mM EDTA, and 5 mg/mL fatty acid-free bovine serum albumin (pH 7.2, 25°C). State 2 respiration was stimulated by the addition of either 5 mM glutamate/5 mM malate or 10 mM succinate and 1 ng/ml rotenone. Transition to state 3 respiration was induced by addition of adenosine diphosphate (ADP, 1 mM). In experiments with isolated mitochondria, 20 μM FeSO_4_ and/or 20 μM desferrioxamine B (Desferal, Novartis, Vienna, Austria) were added to a number of samples before the onset of hypoxia. Mitochondrial function was determined before hypoxia [baseline (BL)], immediately after hypoxia and after 15 min of ROX. To determine mitochondrial function after hypoxia, mitochondria, incubated under hypoxic conditions, were moved in buffer equilibrated with air. Measurements were started immediately and were completed 2 min later.

### Liver enzyme assay

ALT and AST levels were analyzed in the incubation medium after PCLS incubations using a Cobas c 111 reader (Roche, Basel, Switzerland). Samples were taken at different time points as indicated in figure legends.

### cDNA synthesis and RT-PCR

RNA was isolated from snap frozen PCLS using illustra RNAspin Mini RNA Isolation Kit (GE Healthcare, Buckinghamshire, UK). The amount of extracted RNA was determined spectrophotometrically at 260 nm and purity was assessed by the 260/280 nm ratio on an Eppendorf BioPhotometer plusUV/Vis (Eppendorf, Hamburg, Germany). RNA integrity was controlled by microcapillary gel analyzes using Agilent Bioanalyzer 2001 System and RNA6000 nano LabChip Kit (Agilent Technologies, Santa Clara, CA, USA). 1 μg of total RNA was used for cDNA synthesis. cDNA was generated using Superscript™ II RNAse H- reverse transcriptase (200U/reaction; Invitrogen; Carlsbad, CA, USA) and anchored oligo-dT-primers (3.5 μmol/L final concentration). Successful cDNA generation was checked by conventional PCR using GAPDH specific primers, as described elsewhere (Duvigneau et al., 2003). Equal aliquots from each cDNA were pooled to generate an internal standard (IS) which was used as reference for the quantification. Analysis of gene expression was performed by means of qPCR. Primer pairs for the analysis of HO-1 are described elsewhere (Duvigneau et al., 2008). Primer paires for the analysis of IL6 and iNOS were newly designed. Further details regarding settings and validation of the qPCR assays in adhering to MIQE guidelines can be found in the supplementary material (**Table S1**; **Figure S1**). qPCR was carried out on a CFX96™ (Bio-Rad, Hercules, CA, USA). Each reaction contained SYBR® green I as reporter (0.5×, Sigma, USA), iTaq™ polymerase™ (0.625 U/reaction; BioRad), the primers (250 μnmol/l each, Invitrogen) with a final concentration of 200 μmol/l dNTP (each) and 3 mmol/l MgCl_2_ in the provided reaction buffer with a final volume of 12 μ l. All samples were measured in duplicates. Each plate contained corresponding randomly assigned RT-minus controls (10% of all samples investigated), the no-template-control (NTC), as well as the IS. Data were analyzed using the inbuilt software CFX manager (Version 2.0, Bio-Rad) in the linear regression mode. Expression of target genes was calculated against the IS using a modified comparative ΔΔCq method (Schmittgen and Livak, 2008). First the gene specific Cqs were substracted from the mean Cq of the IS obtained for the same gene giving rise to ΔCq. The values were than substracted from the normalization factor, which was calculated by averaging the ΔCqs of the internal reference genes, of the same sample (ΔΔCq). The obtained ΔΔCq values of the replicates were averaged and expressed as 2^-DDCq in fold changes relative to the IS.

### Statistical analysis

Data are displayed as mean ± SEM. All parameters were tested for normality (Kolmogorov–Smirnov test) prior to analysis. Statistical analysis of data was performed by one way ANOVA followed by LSD *post-hoc* test in normally distributed data and Kruskal–Wallis combined with Mann–Whitney test in groups showing a non-Gaussian distribution. Calculations were performed using SPSS 15 software (SPSS Inc., USA). The number of independent samples (*n*) is indicated in figure legends. The significance level was set at 0.05 and is indicated as ^*^p < 0.05, ^**^p < 0.01; ^***^p < 0.001.

## Results

### The effect of HOX/ROX and IM on markers of hepatocyte damage (ALT and AST)

**Figure 1:**
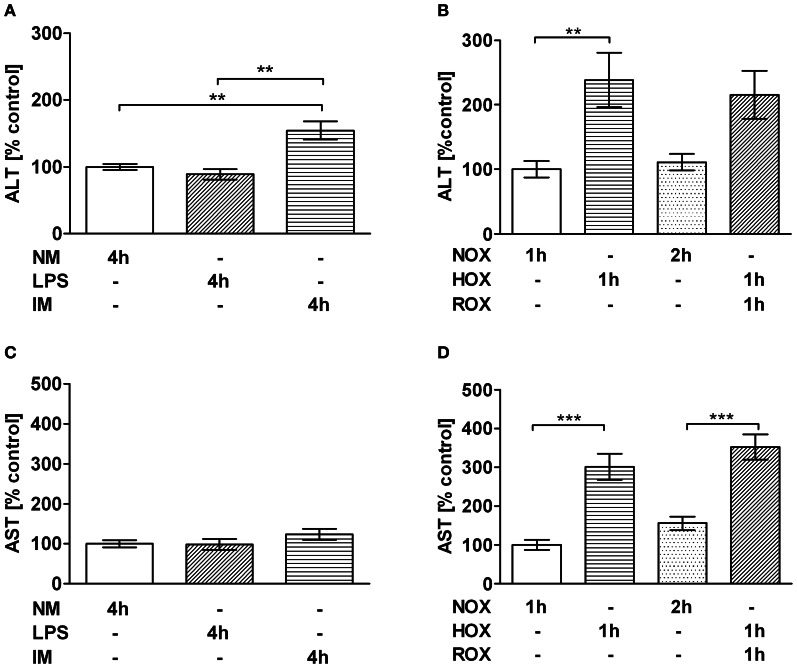
**Effect of IM (A,C) and HOX/ROX (B,D) on the release of ALT (A,B) and AST (C,D) from PCLS. (A,C)** PCLS were incubated in NM only or NM containing LPS or LPS+IM at 37°C. ALT/AST samples were taken after 4 h incubation. **(B,D)** PCLS were incubated in NM which was either equilibrated with air (NOX, ROX) or nitrogen (HOX). The medium for ALT/AST analysis was taken at the end of the hypoxic phase (1 h) and the end of the subsequent reoxygenation phase (1 h HOX + 1 h ROX). Data are expressed as mean ± SEM of at least *n* = 4 and as a percentage of control. ^**^p < 0.01; ^***^p < 0.001. Abbreviations used: ALT, alanine aminotransferase; AST, aspartate aminotransferase; NM, normal medium; LPS, lipopolysaccharide; IM, inflammatory mediators; NOX, Normoxia; HOX, Hypoxia; ROX, reoxygenation; PCLS, precision cut liver slices.

Initially the experimental HOX/ROX-PCLS and IM-PCLS models were standardized. Standardization of the models is based on a significant elevation of ALT release as a basic marker of hepatocyte damage. In the IM-PCLS model significantly elevated ALT levels were observed following 4 h of incubation with IM (Figure 1A), while AST levels remained unchanged (Figure 1C). In the HOX/ROX-PCLS model pronounced and statistically significantly elevated ALT and AST levels were observed after 1 h of hypoxia. No further release of ALT or AST was observed after subsequent ROX (1 h) (Figures 1B,D). To analyze whether IM induced inflammatory response in PCLS, we tested the expression of genes associated with inflammation.

### Changes in gene expression in PCLS following HOX/ROX and incubation with IM

**Figure 2:**
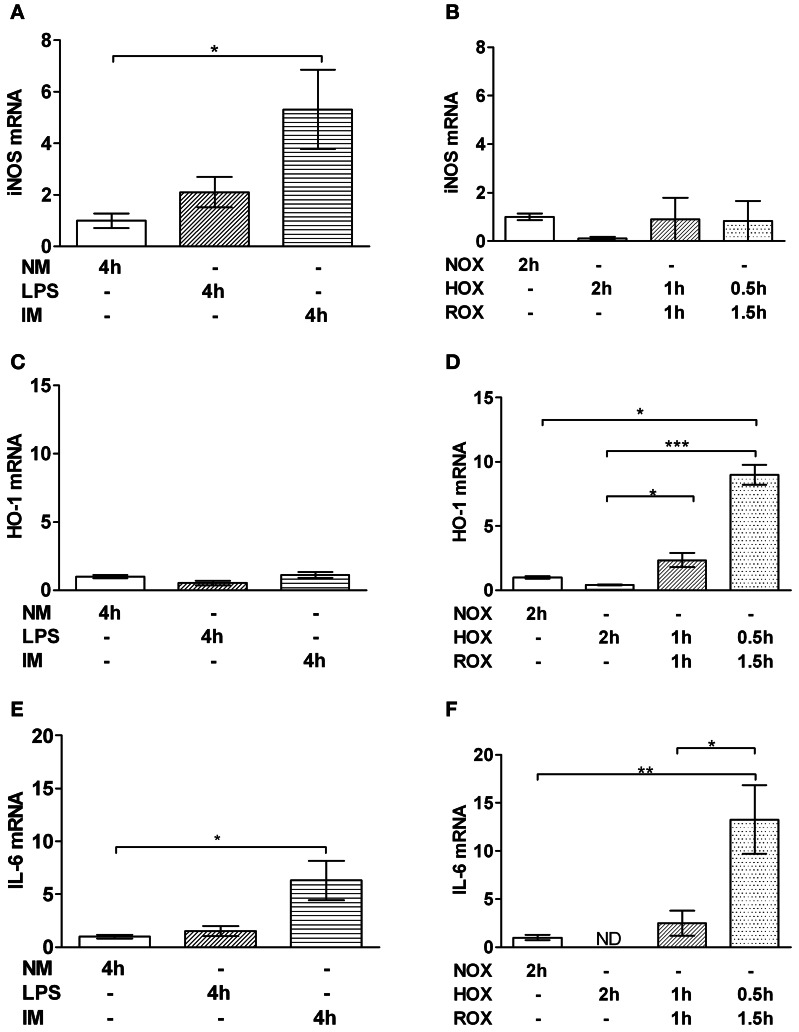
**Effect of IM (A,C,E) and HOX/ROX (B,D,F) on gene expression of iNOS (A,B), HO-1 (C,D) and IL-6 (E,F)**. PCR analysis samples were collected after 4 h of PCLS incubation in NM only or NM containing LPS or LPS + IM at 37°C **(A,C,E)** or after 2 h of PCLS incubation in NM which was either equilibrated with air (NOX, ROX) or nitrogen (HOX) **(B,D,F)**. Data are expressed as mean ± SEM of at least *n* = 4. ^*^p < 0.05; ^**^p < 0.01, ^***^p < 0.001. mRNA expression is represented as fold increase of the corresponding control. Abbreviations used: iNOS, inducible nitric oxide synthase; HO-1, heme oxygenase 1; NM, normal medium; LPS, lipopolysaccharide; IM, inflammatory mediators; NOX, Normoxia; HOX, Hypoxia; ROX, reoxygenation; PCLS, precision cut liver slices; ND, not detectable.

IM led to an upregulation of iNOS (Figure 2A) and IL-6 (Figure 2E) but did not change HO-1 gene expression (Figure 2C). In contrast, in the HOX/ROX model we did not observe any increase in iNOS gene expression (Figure 2B) but HO-1 (Figure 2D) and IL-6 (Figure 2F) were significantly upregulated. A shorter HOX-phase (0.5 h) and longer ROX-phase (1.5 h) increased expression of HO-1 (Figure 2D) and IL-6 (Figure 2F).

### The effect of HOX/ROX and IM on respiratory function of mitochondria in PCLS

**Figure 3:**
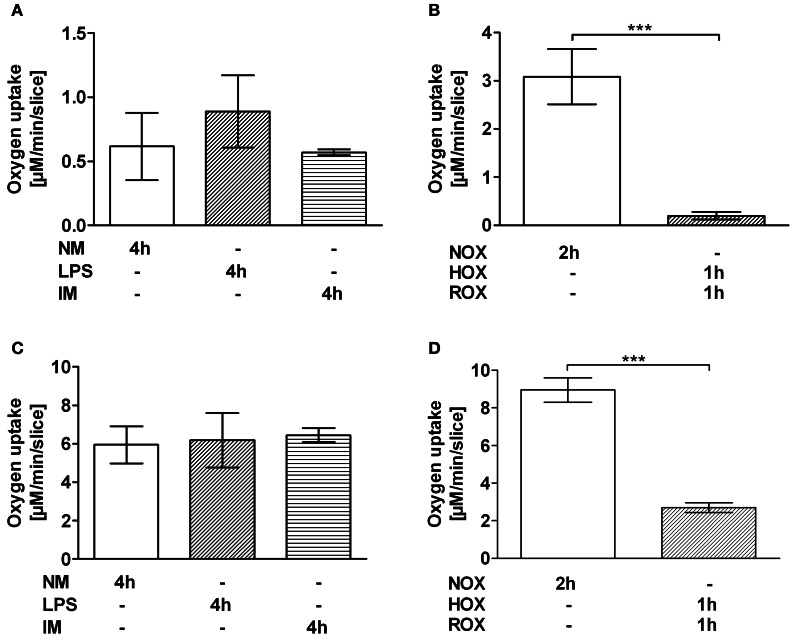
**Effect of IM (A,C) and HOX/ROX (B,D) on the mitochondrial state 3 respiration**. PCLS mitochondria were stimulated with glutamate + malate **(A,B)** or succinate **(C,D)**. Transition to state 3 respiration was induced by addition of ADP (1 mM). Mitochondrial function was determined after 4 h of PCLS incubation in NM only or NM containing LPS or LPS + IM at 37°C **(A,C)** or after 2 h of PCLS incubation in NM which was either equilibrated with air (NOX, ROX) or nitrogen (HOX) **(B,D)**. Data are expressed as mean ± SEM of at least *n* = 4. ^***^p < 0.001. Abbreviations used: NM, normal medium; LPS, lipopolysaccharide; IM, inflammatory mediators; NOX, Normoxia; HOX, hypoxia; ROX, reoxygenation; PCLS, precision cut liver slices.

Incubation with IM did not impair mitochondrial respiration (Figures 3A,C). Incubation with LPS showed a trend to increasing rate of mitochondrial respiration with glutamate/malate, a complex I substrate (Figure 3A). However, HOX/ROX led to a decrease in state 3 respiration, reflecting ATP synthesis, by a factor of 15.4 when mitochondria respired with glutamate/malate (Figure 3B) and by a factor of 3.3 when mitochondria respired with succinate (Figure 3D), a complex II substrate.

### Accumulation of free iron upon hypoxia

**Figure 4:**
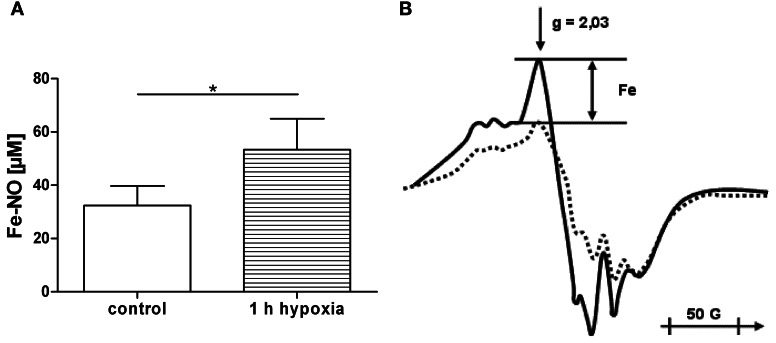
**(A,B)** Accumulation of free iron in liver sections under hypoxia. The sections were treated with nitrite solution and frozen for EPR analysis either immediately after preparation (control) or after incubation for 1 h at 37°C under nitrogen (1 h hypoxia). The difference in free iron concentration between control and 1 h hypoxia was approx. 20 nmol/g tissue **(A)**. The amplitudes (Fe) of the *g* = 2.03 peak were measured in the EPR spectra **(B)** of control liver sections (dotted line) and after 1 h hypoxia (full line) to determine the concentration of nitrosyl iron complexes. Data are expressed as mean ± SEM of *n* = 3. ^*^p < 0.05.

Since iron is known to induce mitochondrial dysfunction, we determined whether or not free iron levels are increased during 1 h of hypoxia. One hour of hypoxia in liver sections resulted in an increase in intracellular free iron levels by approx. 20 nmol/g tissue (Figure 4). In the following experiments we tested the effect of 20 nmol/ml ferrous ion concentration on mitochondrial function.

### The effect of HOX/ROX and iron on respiratory function of isolated rat liver mitochondria

**Figure 5:**
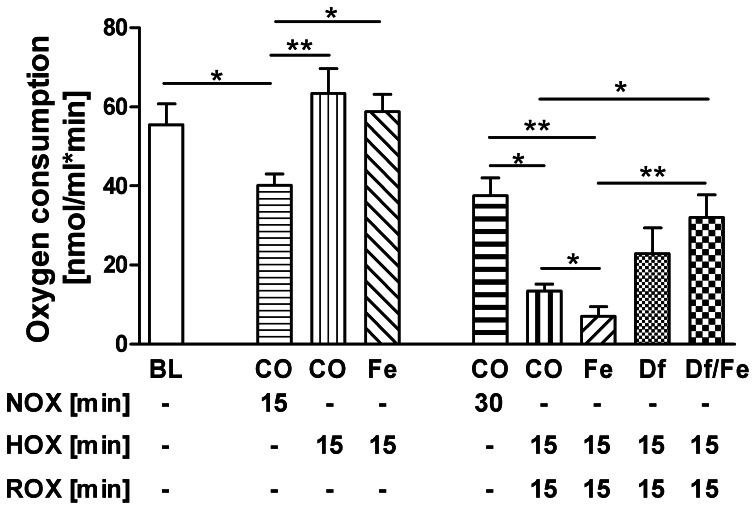
**Effect of HOX, ROX, and free iron on state 3 mitochondrial respiration rate**. The baseline (BL) represents the respiration of freshly isolated mitochondria. Furthermore, mitochondria were incubated either under air (NOX) or under nitrogen (HOX) with 20 μM FeSO_4_ (Fe) or without (CO) for 15 min. Subsequently, all samples were incubated under air for further 15 min (ROX). A group of samples received 20 μM desferrioxamine B (Df) immediately after onset of hypoxia. Data are expressed as mean ± SEM of at least *n* = 5. ^*^p < 0.05; ^**^p < 0.01. Abbreviations used: NOX, Normoxia; HOX, Hypoxia; ROX, reoxygenation; BL, Baseline; CO, control, Fe, FeSO_4_; Df, desferrioxamine B.

To better understand the mechanisms of mitochondrial dysfunction under hypoxic conditions and the impact of free iron, we performed experiments with isolated mitochondria. No decrease of respiratory activity of mitochondria was observed following 15 min of hypoxic conditions (Figure 5). The presence of iron (Fe) in the hypoxic phase did not influence respiratory activity at this time point. However, after 15 min of ROX mitochondrial function was drastically impaired. The addition of iron chelator desferrioxamine B (Df) abolished the difference in respiration rates between samples with and without iron.

## Discussion

The aim of this study was to clarify the impact of two pathophysiologic stressors, IM and HOX/ischemia that typically accompany SIRS, and compromise liver function. Since both stressors are strongly linked to and influenced by each other, their effects cannot be dissected *in vivo*. Most *in vitro* models, however, lack the complexity of liver tissue, and the observed responses may therefore not reflect the *in vivo* situation. In this study we have used PCLS in which liver structures are kept intact, including the different liver cell types. This *in vitro* model allowed us to distinguish between the effects of IM and HOX/ROX on liver cells, since they can be studied separately. Inflammation affects hepatocytes via IM which are formed by activated immune cells in the blood and by Kupffer cells residing in liver tissue. To simulate the effects of systemic inflammation, we used IM generated *ex vivo* by white blood cells treated with LPS. To simulate effects of IM produced by Kupffer cells, we stimulated PCLS directly with LPS. Both models (HOX/ROX and IM) caused damage to liver cells as seen from increased levels of ALT. Examination of AST levels in both models showed an increase only in the HOX model, suggesting mitochondrial damage. Interestingly, in the HOX model both AST and ALT accumulated during the hypoxic phase, while ROX did not change these values. This suggests that the release of these enzymes is not due to reperfusion induced oxidative damage. Alternatively, the damage to biomembranes under hypoxic conditions may be due to deprivation of ATP and subsequent release of calcium ions. Calcium activates phospholipases, which cause the increase in membrane permeability. It has been shown that the inhibition of phospholipase A2 abolishes the release of ALT in dogs subjected to liver ischemia/reperfusion during reperfusion (Ogata et al., 2001). We did not observe any effects of LPS, although the dose of LPS used in our experiments was reported to induce the release of ALT in *in vivo* (Duvigneau et al., 2008). The fact that only the HOX model induced an increase in AST suggests that HOX predominantly causes mitochondrial dysfunction. This assumption was supported by data relating to the respiratory activity of mitochondria in PCLS. IM did not significantly compromise mitochondrial function, while HOX induced a drastic impairment of respiratory activity. Interestingly, this effect was more pronounced in the presence of glutamate/malate, a substrate of complex I, than in the presence of succinate, a substrate of complex II. This is in line with previous publications carried out in *in vivo* models showing a decrease in mitochondrial function upon ischemia/reperfusion (I/R) due to inhibition of complex I and cytochrome c release (Kuznetsov et al., 2004). Mitochondrial dysfunction might be mediated by changes in iron availability, as both iron deficiency and iron excess impair mitochondrial function (Walter et al., 2002). This is in accordance with reports suggesting the involvement of iron in a transient defect in mitochondrial function observed 8 h after challenge with endotoxin (Duvigneau et al., 2008). Moreover, the release of iron has been suggested to be a key event during oxidative mitochondrial damage and hepatocellular injury (Uchiyama et al., 2008). Free iron concentration in fresh liver sections was about 30 nmol/g tissue which is in line with previously published data (Kozlov et al., 1992). It has also been shown that free iron occurs in the ferrous form. Exposure of liver specimens to warm ischemia for 1 h resulted in an increase of free iron levels of 20 nmol/g tissue compared to controls, which is in accordance with previous publications (Sergent et al., 2005). This concentration was used in this study with isolated mitochondria in order to test whether or not this particular concentration of iron can induce the changes we have observed in experiments with PCLS. To clarify whether iron-mediated mitochondrial damage occurs under HOX or during ROX we used equal short-term HOX and ROX induction (15 min each) with or without adding iron ions. State 3 respiration did not change compared to baseline immediately after HOX phase, but was drastically decreased after 15 min of ROX, suggesting that ferrous iron did not impair mitochondrial function under hypoxic conditions. The decrease in state 3 respiration during ROX was more pronounced in the presence of iron ions. The iron chelator desferrioxamine B increased mitochondrial respiration rates to the levels of NOX controls in samples with or without iron. The latter suggests that endogenous iron ions may contribute to the mitochondrial damage. Interestingly, mitochondria in NOX controls had a lower respiration rate than samples subjected to HOX, suggesting that the mechanisms of mitochondrial damage in this model are strictly oxygen dependent. In the IM model we found a significant upregulation of iNOS after 4 h. Others have reported increased iNOS levels after 3 h of LPS stimulation (Olinga et al., 2001). However, the concentration of LPS used in the cited study was 15 times higher than in our study. Coincidence between upregulation of iNOS and increased release of ALT in our suggests, that damage to hepatocyte membrane occurs in this model in association with the immune response, without affecting mitochondria, since neither the mitochondrial function nor AST levels were affected. These data show that iNOS is a very reliable marker for the inflammatory response. Application of HOX/ROX did not result in an upregulation of iNOS. Thus, our data suggest that the activation of iNOS in HOX models previously reported (Duvigneau et al., 2010) is due to secondary inflammatory response rather than due to hypoxia. HO-1 expression was rapidly upregulated only in the HOX model, but not in the IM model, even though the incubation period was longer in the latter model. We conclude that early upregulation of HO-1 is primarily responsive to changes in the redox balance, presumably via HIF-1α and Nrf2. Previous studies documented an upregulation of HO-1 both during SIRS and hypoxia in the liver in different *in vivo* models (Bauer et al., 2003; Duvigneau et al., 2010). Here we show that HO-1-upregulation is oxygen rather than IM dependent. Therefore, the increase of HO-1 mRNA seen under conditions of SIRS, have to be attributed primarily to disturbed oxygen delivery to the target cells, due to hypoxia and an inhibition of mitochondrial function. This suggests that activation of HO-1 in SIRS models is mainly due to secondary circulatory failure rather than due to a direct interaction with IM. The predominant pathway of HO-1 regulation is the Nrf2-dependent pathway. It has been shown that Nrf-2 is upregulated during the post-ischemic ROX phase (Leonard et al., 2006). In addition, it has been shown that NF-kB may also be involved in the upregulation of HO-1, although these data are still controversial [reviewed in Paine et al. (2010)].

In contrast to iNOS and HO-1 which were upregulated only in one of the two models, IL-6 responded to both hypoxia and IM. This can be explained by the fact that numerous signaling pathways are known to activate IL-6 mRNA expression, via NF-kB, JNK, C/EBP/(NF)-IL6 and CREB-dependent pathways. It has already been shown that hypoxia induces the (NF)-IL-6 pathway (Yan et al., 1995). In addition, IL-6 may be upregulated by a number of IM via NF-kB-dependent pathway similarly to iNOS (Hur et al., 1999; Li and Verma, 2002). Thus, IL-6 synthesis may be activated by both IM and impaired oxygen delivery. Shortening of the HOX-phase (0.5 h) and extension of the ROX-phase (1.5 h) strongly increased expression of both HO-1 and IL-6, suggesting that either HOX-phase inhibits or ROX-phase activates gene expression most likely through decreased ATP levels.

In summary, in this study we distinguished between the pathological impacts of hypoxia and direct interaction of IM with parenchymal cells in PCLS based models. These *in vitro* models can be used to analyze other parameters accompanying either SIRS or circulatory failure. In addition our data show that accumulation of free iron in hypoxic tissue is critical for mitochondrial function. This is in line with previous reports showing beneficial effects of iron chelators in hypoxia models (Arkadopoulos et al., 2010) and sepsis models (Srinivasan et al., 2012). A clear understanding of whether HOX or IM dependent pathways predominantly cause organ failure in certain pathologic states will contribute to the development of new effective therapy approaches.

The major limitation of this study is that experiments were performed at a defined set of conditions of either hypoxia (e.g., duration of HOX and ROX) or inflammation (e.g., concentration of IM and incubation time). The balance between HOX-dependent and IM-dependent processes may depend on the severity of a disease or an experimental model.

### Conflict of interest statement

The authors declare that the research was conducted in the absence of any commercial or financial relationships that could be construed as a potential conflict of interest.

## References

[B1] Arkadopoulos N. Nastos C. Kalimeris K. Economou E. Theodoraki K. Kouskouni E. (2010). Iron chelation for amelioration of liver ischemia-reperfusion injury. Hemoglobin 34, 265–277 10.3109/03630269.2010.484766 20524816

[B2] Bauer I. Rensing H. Florax A. Ulrich C. Pistorius G. Redl H. (2003). Expression pattern and regulation of heme oxygenase-1/heat shock protein 32 in human liver cells. Shock 20, 116–122 10.1097/01.shk.0000075568.93053.fa 12865654

[B3] Clarkson A. N. Sutherland B. A. Appleton I. (2005). The biology and pathology of hypoxia-ischemia: an update. Arch. Immunol. Ther. Exp. 53, 213–225 15995582

[B4] Dungel P. Haindl S. Behling T. Mayer B. Redl H. Kozlov A. V. (2011). Neither nitrite nor nitric oxide mediate toxic effects of nitroglycerin on mitochondria. J. Biochem. Mol. Toxicol. 25, 297–302 10.1002/jbt.20389 21523859

[B5] Duvigneau J. C. Hartl R. T. Teinfalt M. Gemeiner M. (2003). Delay in processing porcine whole blood affects cytokine expression. J. Immunol. Methods 272, 11–21 10.1016/S0022-1759(02)00372-1 12505708

[B6] Duvigneau J. C. Kozlov A. V. Zifko C. Postl A. Hartl R. T. Miller I. (2010). Reperfusion does not induce oxidative stress but sustained endoplasmic reticulum stress in livers of rats subjected to traumatic-hemorrhagic shock. Shock 33, 289–298 10.1097/SHK.0b013e3181aef322 19503022

[B7] Duvigneau J. C. Piskernik C. Haindl S. Kloesch B. Hartl R. T. Hüttemann M. (2008). A novel endotoxin-induced pathway: upregulation of heme oxygenase 1, accumulation of free iron, and free iron-mediated mitochondrial dysfunction. Lab. Invest. 88, 70–77 10.1038/labinvest.3700691 17982471

[B8] Hur G. M. Ryu Y. S. Yun H. Y. Jeon B. H. Kim Y. M. Seok J. H. (1999). Hepatic ischemia/reperfusion in rats induces iNOS gene transcription by activation of NF-kappaB. Biochem. Biophys. Res. Commun. 261, 917–922 10.1006/bbrc.1999.1143 10441525

[B9] Isobe M. Katsuramaki T. Hirata K. Kimura H. Nagayama M. Matsuno T. (1999). Benefical effects of inducible nitric oxide synthase inhibitor on reperfusion injury in the pig liver. Transplantation 68, 803–813 10515381 10.1097/00007890-199909270-00013

[B10] Izeboud C. A. Hoebe K. H. N. Grootendorst A. F. Nijmeijer S. M. Van Miert A. S. Witkamp R. R. (2004). Endotoxin-induced liver damage in rats is minimized by beta 2-adrenoceptor stimulation. Inflamm. Res. 53, 93–99 10.1007/s00011-003-1228-y 15021963

[B11] Kielar M. L. John R. Bennett M. Richardson J. A. Shelton J. M. Chen L. (2005). Maladaptive role of IL-6 in ischemic acute renal failure. J. Am. Soc. Nephrol. 16, 3315–3325 10.1681/ASN.2003090757 16192425

[B12] Kozlov A. V. Yegorov D. Y. Vladimirov Y. A. Azizova O. A. (1992). Intracellular free iron in liver tissue and liver homogenate: studies with electron paramagnetic resonance on the formation of paramagnetic complexes with desferal and nitric oxide. Free Radic. Biol. Med. 13, 9–16 10.1016/0891-5849(92)90159-E 1321074

[B13] Kuznetsov A. V. Schneeberger S. Seiler R. Brandacher G. Mark W. Steurer W. (2004). Mitochondrial defects and heterogeneous cytochrome c release after cardiac cold ischemia and reperfusion. Am. J. Physiol. Heart Circ. Physiol. 286, H1633–H1641 10.1152/ajpheart.00701.2003 14693685

[B14] Legrand M. Klijn E. Payen D. Ince C. (2010). The response of the host microcirculation to bacterial sepsis: does the pathogen matter? J. Mol. Med. 88, 127–133 10.1007/s00109-009-0585-6 20119709 PMC2832870

[B15] Leonard M. O. Kieran N. E. Howell K. Burne M. J. Varadarajan R. Dhakshinamoorthy S. (2006). Reoxygenation-specific activation of the antioxidant transcription factor Nrf2 mediates cytoprotective gene expression in ischemia-reperfusion injury. FASEB J. 20, 2624–2626 10.1096/fj.06-5097fje 17142801

[B16] Lerche-Langrand C. Toutain H. J. (2000). Precision-cut liver slices: characteristics and use for *in vitro* pharmaco-toxicology. Toxicology 153, 221–253 10.1016/S0300-483X(00)00316-4 11090959

[B17] Li Q. Verma I. M. (2002). NF-kappaB regulation in the immune system. Nat. Rev. Immunol. 2, 725–734 10.1038/nri910 12360211

[B18] Lin T.-T. Wang B.-M. Li X.-Y. Pan Y. Wang W. Mu Y. (2009). An insight into the protection of rat liver against ischemia/reperfusion injury by 2-selenium-bridged beta-cyclodextrin. Hepatol. Res. 39, 1125–1136 10.1111/j.1872-034X.2009.00545.x 19624763

[B19] Murphy M. P. (2009). How mitochondria produce reactive oxygen species. Biochem. J. 417, 1–13 10.1042/BJ20081386 19061483 PMC2605959

[B20] Ogata K. Jin M. B. Taniguchi M. Suzuki T. Shimamura T. Kitagawa N. (2001). Attenuation of ischemia and reperfusion injury of canine livers by inhibition of type II phospholipase A2 with LY329722. Transplantation 71, 1040–1046 11374398 10.1097/00007890-200104270-00004

[B21] Olinga P. Merema M. T. De Jager M. H. Derks F. Melgert B. N. Moshage H. (2001). Rat liver slices as a tool to study LPS-induced inflammatory response in the liver. J. Hepatol. 35, 187–194 11580140 10.1016/s0168-8278(01)00103-9

[B22] Paine A. Eiz-Vesper B. Blasczyk R. Immenschuh S. (2010). Signaling to heme oxygenase-1 and its anti-inflammatory therapeutic potential. Biochem. Pharmacol. 80, 1895–1903 10.1016/j.bcp.2010.07.014 20643109

[B23] Rouslin W. Broge C. W. Grupp I. L. (1990). ATP depletion and mitochondrial functional loss during ischemia in slow and fast heart-rate hearts. Am. J. Physiol. 259, H1759–H1766 2148059 10.1152/ajpheart.1990.259.6.H1759

[B24] Schmittgen T. D. Livak K. J. (2008). Analyzing real-time PCR data by the comparative CT method. Nat. Protoc. 3, 1101–1108 18546601 10.1038/nprot.2008.73

[B25] Sergent O. Tomasi A. Ceccarelli D. Masini A. Nohl H. Cillard P. (2005). Combination of iron overload plus ethanol and ischemia alone give rise to the same endogenous free iron pool. Biometals 18, 567–575 10.1007/s10534-005-8488-7 16388396

[B26] Shiva S. Sack M. N. Greer J. J. Duranski M. Ringwood L. A. Burwell L. (2007). Nitrite augments tolerance to ischemia/reperfusion injury via the modulation of mitochondrial electron transfer. J. Exp. Med. 204, 2089–2102 10.1084/jem.20070198 17682069 PMC2118713

[B27] Singer M. De Santis V. Vitale D. Jeffcoate W. (2004). Multiorgan failure is an adaptive, endocrine-mediated, metabolic response to overwhelming systemic inflammation. Lancet 364, 545–548 10.1016/S0140-6736(04)16815-3 15302200

[B28] Skulachev V. P. (1999). Mitochondrial physiology and pathology; concepts of programmed death of organelles, cells and organisms. Mol. Aspects Med. 20, 139–184 10.1016/S0098-2997(99)00008-4 10626278

[B29] Srinivasan G. Aitken J. D. Zhang B. Carvalho F. A. Chassaing B. Shashidharamurthy R. (2012). Lipocalin 2 deficiency dysregulates iron homeostasis and exacerbates endotoxin-induced sepsis. J. Immunol. 189, 1911–1919 10.4049/jimmunol.1200892 22786765 PMC3411903

[B30] Tacchini L. Cairo G. De Ponti C. Massip M. Rosellò-Catafau J. Peralta C. (2006). Up regulation of IL-6 by ischemic preconditioning in normal and fatty rat livers: association with reduction of oxidative stress. Free Radic. Res. 40, 1206–1217 10.1080/10715760600885432 17050174

[B31] Tenhunen R. Marver H. S. Schmid R. (1968). The enzymatic conversion of heme to bilirubin by microsomal heme oxygenase. Proc. Natl. Acad. Sci. U.S.A. 61, 748–755 4386763 10.1073/pnas.61.2.748 PMC225223

[B32] Uchiyama A. Kim J.-S. Kon K. Jaeschke H. Ikejima K. Watanabe S. (2008). Translocation of iron from lysosomes into mitochondria is a key event during oxidative stress-induced hepatocellular injury. Hepatology 48, 1644–1654 10.1002/hep.22498 18846543 PMC2579320

[B33] Walter P. B. Knutson M. D. Paler-Martinez A. Lee S. Xu Y. Viteri F. E. (2002). Iron deficiency and iron excess damage mitochondria and mitochondrial DNA in rats. Proc. Natl. Acad. Sci. U.S.A. 99, 2264–2269 10.1073/pnas.261708798 11854522 PMC122353

[B34] West A. P. Brodsky I. E. Rahner C. Woo D. K. Erdjument-Bromage H. Tempst P. (2011). TLR signalling augments macrophage bactericidal activity through mitochondrial ROS. Nature 472, 476–480 10.1038/nature09973 21525932 PMC3460538

[B35] Yan S. F. Tritto I. Pinsky D. Liao H. Huang J. Fuller G. (1995). Induction of interleukin 6 (IL-6) by hypoxia in vascular cells. J. Biol. Chem. 270, 11463–11471 7744784 10.1074/jbc.270.19.11463

