# Supplementary material for: Experimental data suggesting that inflammation mediated rat liver mitochondrial dysfunction results from secondary hypoxia rather than from direct effects of inflammatory mediators
Source: Front Physiol. 2013 Jun 7;4:138. doi: 10.3389/fphys.2013.00138 (PMC3675332; doi:10.3389/fphys.2013.00138)
Supplement: Supplementary file 1 [file Presentation1.PPT]

## Slide 1
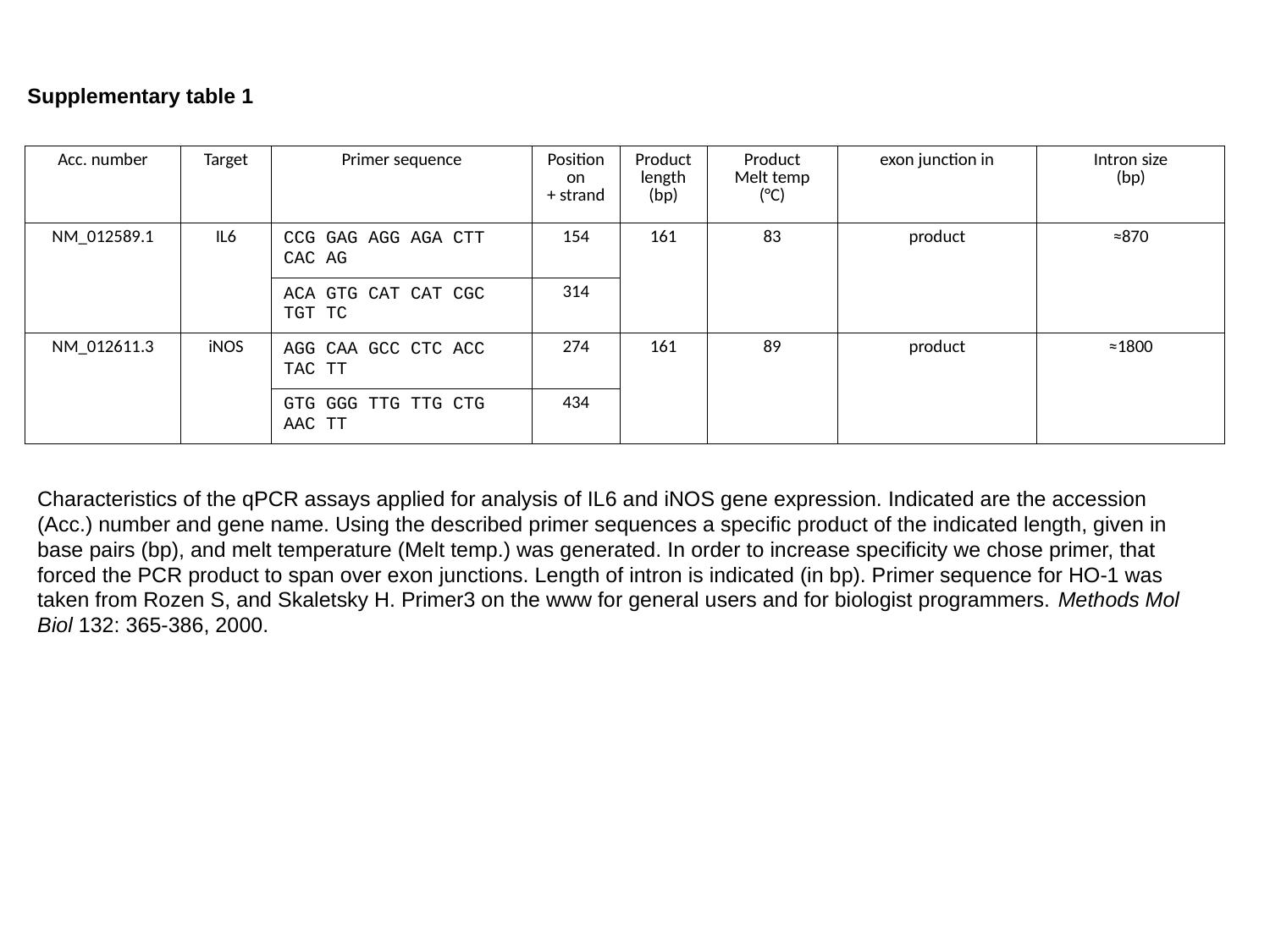

Supplementary table 1
| Acc. number | Target | Primer sequence | Position on + strand | Product length (bp) | Product Melt temp (°C) | exon junction in | Intron size (bp) |
| --- | --- | --- | --- | --- | --- | --- | --- |
| NM\_012589.1 | IL6 | CCG GAG AGG AGA CTT CAC AG | 154 | 161 | 83 | product | ≈870 |
| | | ACA GTG CAT CAT CGC TGT TC | 314 | | | | |
| NM\_012611.3 | iNOS | AGG CAA GCC CTC ACC TAC TT | 274 | 161 | 89 | product | ≈1800 |
| | | GTG GGG TTG TTG CTG AAC TT | 434 | | | | |
Characteristics of the qPCR assays applied for analysis of IL6 and iNOS gene expression. Indicated are the accession (Acc.) number and gene name. Using the described primer sequences a specific product of the indicated length, given in base pairs (bp), and melt temperature (Melt temp.) was generated. In order to increase specificity we chose primer, that forced the PCR product to span over exon junctions. Length of intron is indicated (in bp). Primer sequence for HO-1 was taken from Rozen S, and Skaletsky H. Primer3 on the www for general users and for biologist programmers. Methods Mol Biol 132: 365-386, 2000.

## Slide 2
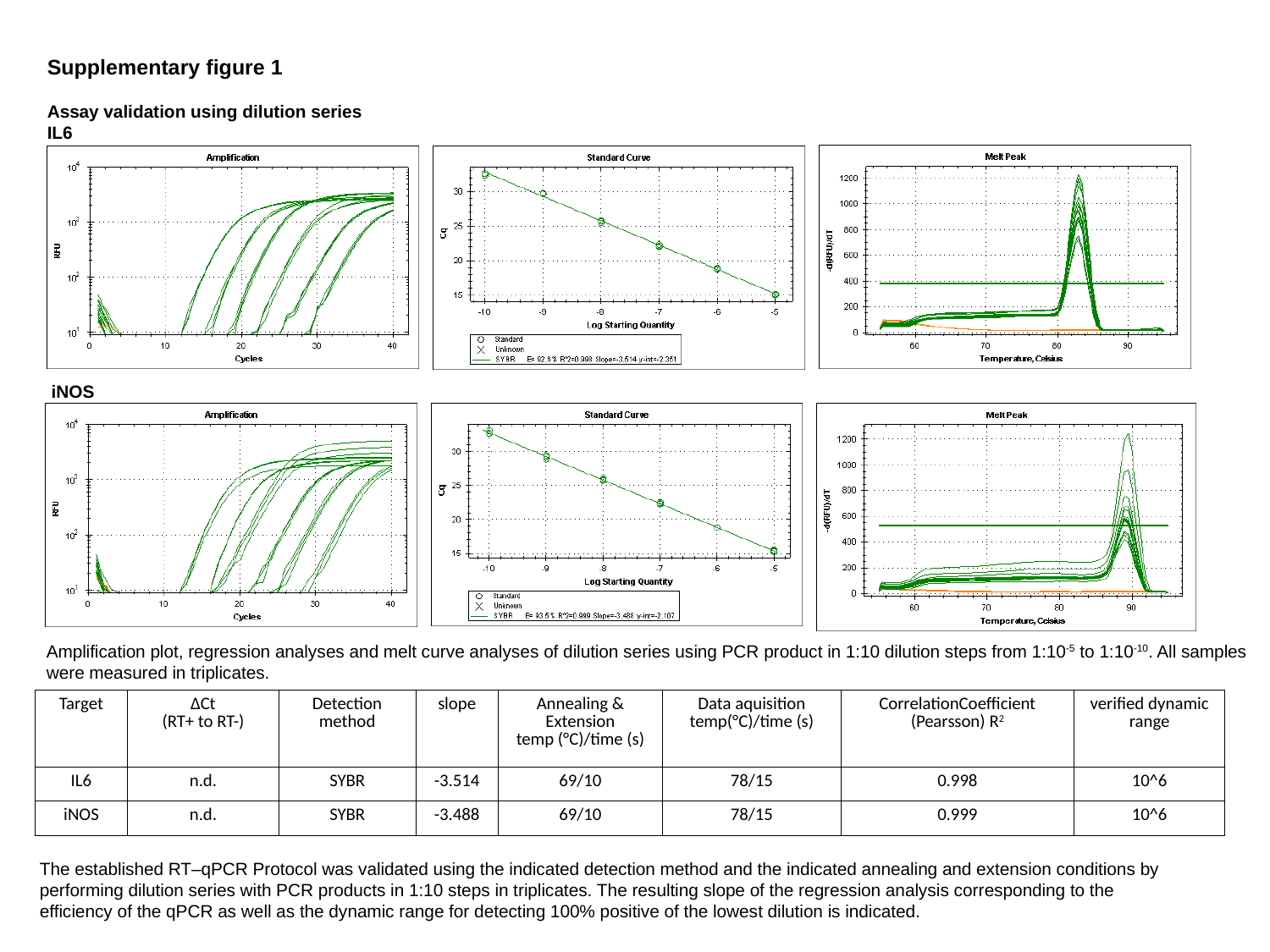

Supplementary figure 1
Assay validation using dilution series
IL6
iNOS
Amplification plot, regression analyses and melt curve analyses of dilution series using PCR product in 1:10 dilution steps from 1:10-5 to 1:10-10. All samples were measured in triplicates.
| Target | ∆Ct (RT+ to RT-) | Detection method | slope | Annealing & Extension temp (°C)/time (s) | Data aquisition temp(°C)/time (s) | CorrelationCoefficient (Pearsson) R2 | verified dynamic range |
| --- | --- | --- | --- | --- | --- | --- | --- |
| IL6 | n.d. | SYBR | -3.514 | 69/10 | 78/15 | 0.998 | 10^6 |
| iNOS | n.d. | SYBR | -3.488 | 69/10 | 78/15 | 0.999 | 10^6 |
The established RT–qPCR Protocol was validated using the indicated detection method and the indicated annealing and extension conditions by performing dilution series with PCR products in 1:10 steps in triplicates. The resulting slope of the regression analysis corresponding to the efficiency of the qPCR as well as the dynamic range for detecting 100% positive of the lowest dilution is indicated.
